# The KTx360°-study: a multicenter, multisectoral, multimodal, telemedicine-based follow-up care model to improve care and reduce health-care costs after kidney transplantation in children and adults

**DOI:** 10.1186/s12913-017-2545-0

**Published:** 2017-08-23

**Authors:** L Pape, M de Zwaan, U Tegtbur, F Feldhaus, JK Wolff, L Schiffer, C Lerch, N Hellrung, V Kliem, G Lonnemann, HD Nolting, M Schiffer

**Affiliations:** 1 Department of Pediatric Kidney, Liver and Metabolic Diseases, Hannover Medical School, Carl-Neuberg-Straße 1, D-30625 Hannover, Germany; 2 Department of Psychosomatic Medicine and Psychotherapy, Hannover Medical School, Hannover, Germany; 3 Department of Sports Medicine, Hannover Medical School, Hannover, Germany; 4 Department of Business Operations, Innovation and Quality Management, Hannover Medical School, Hannover, Germany; 5 IGES Insitute for Health Care Research, Berlin, Germany; 6 Department of Nephrology and Hypertension, Hannover Medical School, Hannover, Germany; 7 Symeda GmbH, Braunschweig, Germany; 8 Department of Nephrology, Nephrologic Center Hannoversch Münden, Hannoversch Münden, Germany; 9 Dialysis Center Eickenhof, Langenhagen, Germany

**Keywords:** Kidney transplantation, Transition, Telemedicine, Case management, Physical exercise, Psychosomatics, Adherence, Cardiovascular risk, Graft survival, Patient survival, Video consultation

## Abstract

**Background:**

Follow-up care after kidney transplantation is performed in transplant centers as well as in local nephrologist’s practices in Germany. However, organized integrated care of these different sectors of the German health care system is missing. This organizational deficit as well as non-adherence of kidney recipients and longterm cardiovascular complications are major reasons for an impaired patient and graft survival.

**Methods:**

The KTx360° study is supported by a grant from the Federal Joint Committee of the Federal Republic of Germany.

The study will include 448 (39 children) incident patients of all ages with KTx after study start in May 2017 and 963 (83 children) prevalent patients with KTx between 2010 and 2016.

The collaboration between transplant centers and nephrologists in private local practices will be supported by internet-based case-files and scheduled virtual visits (patient consultation via video conferencing). At specified points of the care process patients will receive cardiovascular and adherence assessments and respective interventions. Care will be coordinated by an additional case management.

The goals of the study will be evaluated by an independent institute using claims data from the statutory health insurances and data collected from patients and their caregivers during study participation. To model longitudinal changes after transplantation and differences in changes and levels of immunosuppresive therapy after transplantation between study participants and historical data as well as data from control patients who do not participate in KTx360°, adjusted regression analyses, such as mixed models with repeated measures, will be used. Relevant confounders will be controlled in all analyses.

**Discussion:**

The study aims to prolong patient and graft survival, to reduce avoidable hospitalizations, co-morbidities and health care costs, and to enhance quality of life of patients after kidney transplantation.

**Trial registration:**

ISRCTN29416382 (retrospectively registered on 05.05.2017)

## Background

Kidney transplantation (KTx) is the therapy of choice for patients with end stage renal disease and is associated with significantly reduced morbidity and mortality compared to dialysis.

In the first 3 years after KTx about 8% of the patients lose their graft. This increases steadily over the following years [1]. The main reasons for graft loss are chronic rejections and death with functioning graft due to cardiovascular events and non-adherence to the prescribed medication regimen. Several cohort studies by us and others confirmed that about one third of graft losses per year are due to non-adherence [2–4]. Adolescents experience a significant increase of graft failure during the transition phase to adult care.

Currently, post-transplant care in Germany is split between outpatient services of the hospital transplant centres and (pediatric) nephrologists in private practices usually located close to the patient’s place of residence. KTx360° aims to establish a structured post-transplant care program integrating health care providers from different sectors of the German health system including hospital transplant centers, (pediatric) nephrologists in private practice, and other health professionals relevant for patient care such as transplant nurses, psychologists or sports medicine specialists.

## Methods/design

Inclusion criteria for this study are: KTx from 2010 to 2019 that was performed at one of the two kidney transplantation centres in the German state of Lower Saxony, study participation of the (pediatric) nephrologist responsible for the patient’s follow-up, and membership of the patient in a participating statutory health insurance. Patients of all ages can be included.

Two cohorts of patients will be included in KTx360°: incident and prevalent patients. Incident patients will have their kidney transplantation during the recruitment period of the study (2017 to 2019) at one of the two transplant centers in Lower Saxony. There are about 225 kidney transplantations per year in these centers (210 adults and 15 children/adolescents). Assuming a response rate of 80% caused by non-participation of health insurances, private practice nephrologists or patients who decline participation, we expect to include a total sample of *n* = 487 (*n* = 448 adults, *n* = 39 children/ adolescents) incident patients during the 31 months recruitment period. With a rolling recruitment process and an assumed 15% drop-out rate per year due to graft loss, death or withdrawal from the study we expect to obtain data from 286 adults and 26 children/adolescents completing 2 years post-transplant observation and of 121 adults and 12 children/adolescents with three post-transplant years. Prevalent patients who had their kidney transplantation between 2010 and 2016 will be included in the study when they attend their regular follow-up care visits at the transplant centers. Under similar conditions as the incident patients and an assumed 5% mortality rate per year for adults before study inclusion, we expect a total of *n* = 1046 prevalent patients (*n* = 83 children/adolescents) to participate in the study.

Study patients can be compared with two different control groups based on claims data. The first control sample comprises historical data of the prevalent patients enabling comparisons between post-transplant care before and after implementation of KTx360° for patients of the two kidney transplant centres in Lower Saxony (historical comparison group). Second, all participating health insurance companies will provide claims data of their insurants who received a kidney transplantation in a non-participating German transplant centre between 2010 and 2019 (external comparison group). In Germany, there are 2074 kidney transplantations per year [5] with about 5% of transplantations in children or adolescents under the age of 20 years. Taking into account 10% of patients with private health insurances, a response rate of 70% (because not all statutory insurances are participating) and a mortality rate of 5% per year for adults, results in available data of about 12,413 patients in their first year after transplantation. Subtracting the number of study participants (*n* = 1533), we expect to receive claims data on about *n* = 11,533 patients with kidney transplantations in the external comparison group.

An a-priori power analysis for the primary outcome of a 20% reduction of health care costs due to hospitalizations (see section on endpoints below) was conducted based on data of one statutory health insurance. According to these data, average costs of hospital treatments in the first 2 years after transplantation are estimated 19,000 € per patient. The power analysis for the effect of a 20% reduction (two-sided test) was performed in SAS 9.3 under the following assumptions: pooled standard deviation of 10,000, normal distribution, and α = .05. With the expected sample size in year one and two after transplantation of *n* = 286 incident adults, a power of .995 was estimated to find the assumed difference. Thus, we expect sufficient power to detect the hypothesized effect.

**Fig. 1:**
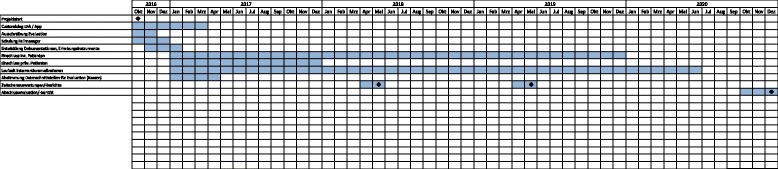
Gantt-Chart showing phases of the study

The main focus of the trial is the long-term improvement in post-transplant patient management by the introduction of eHealth elements and additional integrated therapeutic options. With the involvement of case managers we intend to facilitate the cooperation between transplant centers, nephrologists in private practice, and patients. The establishment of an internet-based case file that can be used by all three parties should further optimize communication, cooperation, and thereby post-transplant care. Especially the possibility for our patients to have personal access to their health data is an innovation that will enhance transparency and self-management abilities. Substituting several face-to-face follow-up visits at the transplantation centres by video consultations in the offices of the (pediatric) nephrologists is supposed to reduce travel costs and may avoid doubling of lab examinations. Based on risk assessments, specific interventions will be offered that enhance health behavior and improve adherence to immunosuppressant medication (IS) and reduce cardiovascular risk. The study design is summarized in Fig. 1.

KTx 360° consists of the following interventions and treatment modules:

### Video consultations

In addition to regular outpatient consultations, video consultations will take place in cooperation between the transplant center and the (pediatric) nephrologist in private practice reducing the number of appointments at the transplant center. At the time of the patient’s appointment at the nephrologist’s office, an experienced physician from the transplant center will be connected to the nephrologist’s office via video platform. Treatment consequences can be discussed together during the session. In case of unexpected medical problems, unscheduled video consultations are possible.

### Psychosomatic-psychosocial risk assessments and adherence coaching via video chat or face-to-face consultations

Even though adherence to immunosuppressants (IS) is crucial to prevent allograft failures, a substantial proportion of transplant recipients are non-adherent to their IS regimen. Among renal transplant patients on average 35.6% of patients per year have been reported to be non-adherent to IS medication [3, 4]. After KTx it is not sufficient to only take the prescribed IS; the timing of IS intake is equally important. Even minor deviations from the prescribed regimen are associated with a higher risk of graft loss. Poor adherence to IS treatment is still the leading preventable cause of graft loss [6].

In our program, each patient will repeatedly receive psychological adherence assessments to screen for non-adherence to IS and to diagnose specific non-adherence phenotypes or patterns. Early in the post-transplant period we will begin with four assessments during the first year and two assessments in the following years. Non-adherence can occur early after transplantation and has been shown to increase over time. Thus, early and continued assessments and interventions after transplantation are considered necessary.

Adherence will be assessed in personal interviews using the Basel Assessment of Adherence with Immunosuppressive Medication Scale (BAASIS©) [7]. A collaborative rather than confrontational interview style will be used in order to elicit honest replies and to avoid social desirability. Non-adherent behaviors broadly fall into two categories: unintentional and intentional nonadherence. Thus, we will assess the following potential barriers of adherence using established instruments: cognitive dysfunctions, lack of resources, lack of knowledge regarding IS, experience of adverse events, mental disorders, psychological distress, low quality of life, and low social support. Finally, applying the necessity-concerns-framework the patients’ personal beliefs about the necessity of their IS medication and their concerns about taking them will be assessed [8].

Depending on the results of the adherence assessments, the psychosocial team will make different recommendations. A patient in need of more information about IS will be referred to an educational group offered by the case managers. It is well known that repetitive teaching is necessary to ensure long-term adherence [9]. Patients can also be referred to a psychologist within the psychosocial team who will offer up to eight treatment sessions per year without waiting period. This intervention can be conducted face-to-face or via video and will deal with psychosocial correlates of non-adherence (e.g., social or work problems, psychological distress) and offer behavioral interventions. If necessary, patients will be referred to more intensive regular care outside the program.

### Cardiovascular assessment and sports therapy via video chat and face-to-face consultations

Exercise capacity after KTx is reduced to 50–70% of normal values [10]. Reasons for reduced physical capacity after KTx are a long period of inactivity during chronic renal failure, side effects of IS such as atrophy of skeletal muscles and osteoporosis, reduced cardiovascular and endothelial function, and lack of long-term rehabilitation programs [11, 12]. Randomized studies have shown that exercise training programs significantly improve maximum oxygen consumption, quality of life, cardiovascular risk, blood pressure, endothelial function and osteoporosis in patients after solid organ transplantation [10, 13, 14]. The magnitude of exercise training effects depends on the duration and the type of the exercise training program.

After KTx in our center, patients usually perform inpatient rehabilitation for 3 weeks directly after hospital discharge. Despite the impressive effects of exercise training in transplant recipients no further intervention to improve daily life activities and physical function are established in long-term rehabilitation in Germany.

Exercise recommendations in transplant recipients include endurance training 2–4 times per week (improving vascular function, reduced cardiovascular risk) and regular resistance training (to prevent osteoporosis and skeletal muscle loss) [15]. In KTx360° a regular home based exercise training program will be implemented. Initiated 2–3 months after transplantation patients (including criteria are duration of dialysis longer than 3 years, coronary artery disease, diabetes mellitus, BMI > 35, patients under the age of 18 years) will perform a physical examination and an incremental exercise testing on an ergometer including blood lactate measurements for determination of cardiovascular and skeletal muscle function. Furthermore, a 30 min constant load test on a cycle ergometer with ECG, blood pressure, blood lactate and glucose monitoring, a Timed up and go test, a Sit-to-Stand-test and measurement of steps per day will be performed initially and every 12 months for evaluation of secondary outcome parameters. Exercise training recommendations are derived from exercise intervention studies with heart and renal transplant patients and include moderate endurance training or moderate resistance training every other day:

- Exercise capacity near to normal (>80% of normal values): Initial duration 15-30 min with 35–60% of maximum exercise capacity.
- Exercise capacity reduced (60–80% of normal values): Initial duration 10-20 min with 30–50% of maximum exercise capacity.
- Exercise capacity reduced (<60% of normal values): Initial duration 15-20 min with 25–40% of maximum exercise capacity.

Exercise training will be monitored by an exercise physiologist via a wearable system to measure physical activities and the respective heart rates. A regular feed–back, based on continuous training data interpretation, will be given monthly by video/phone conference in order to motivate the patient and adapt the training prescriptions. In the first year after KTx patients will be seen 4 times face-to-face (2 times a year thereafter), and during these occasions patients will be monitored during a 30 min endurance exercise training.

### Case management and coordination of post-transplant care/quality management

In the participating transplant centers, a coordination office will be established. Case managers will coordinate post-transplant care individually and offer continuous support to patients. The case managers will have the following tasks: individual organization of post-transplant care including scheduling of follow-up appointments, medical information management, updating the internet-based case file, liaising with regional care partners, organization of regular care outside the program, and preparation as well as coordination of case conferences. Another central task is organization and support of transition from pediatric into adult care. The case management will also organize weekly case conferences (nephrologists, pediatric nephrologists, sports medicine specialists, psychologists, case managers) within the transplant centers and yearly quality circles of all program participants to discuss and decide on standard operating procedures to be followed by all participating care givers.

### Internet-based case file and video consultation platform

The internet-based case-file platform that is based on a customized version of the program “Case Plus®”, provided by the company Symeda GmbH, will be used for documentation of all relevant medical data. In addition, the patient can include daily updates on his/her health status in the internet-based case file. Lab values and other important patient data will regularly be included in the internet-based case file by the case managers, the private practice nephrologists and/or by automatic interfaces to hospital and office software. The internet-based case file will be used for coordinating post-transplant care, documenting patient contacts and utilizing the treatment modules of KTx360°. Data can be exported from the case file and pseudonymized for evaluation purposes. Most of the entries in the internet-based case file can be accessed by the patients at any time.

### Goals

The main goals of KTx360° are an improvement of graft survival and quality of life after kidney transplantation and a reduction of health care costs caused by avoidable hospital treatments due to complications or co-morbidities.

To achieve these goals, sustainable efforts to reduce cardiovascular risks and immunological risks, to increase adherence to medication and scheduled visits, and to enhance care in the transition phase have been implemented in KTx360°. Thereby, we expect to reduce health care costs by reducing the frequency of hospitalizations and by postponing the need for dialysis following graft loss. Due to an individualized psychosocial care, an improvement in quality of life can be expected.

### Study endpoints

The primary endpoint of the study will be the 20% reduction in health care costs due to a reduction of hospital utilization of study participants as compared to the control groups (endpoint 3).

1. Increase of adherence for outpatient visits. Goal: Participation in 90% of scheduled visits.
2. Increase of adherence to immunosuppressive therapy. Goals: 1) Coefficient of variation of levels of immunosuppressants <0.4 in 75% of patients. 2) Non-adherence in BAASIS-interview <10% in incident and <20% in prevalent patients / year.
3. Reduction of transplant-associated complications. Goals: 1) Reduction of hospitalization costs by 20% as compared to a control group (primary endpoint) 2) Reduction of graft losses by 25% as compared to a control group.
4. Improvement of cardiovascular fitness and stabilization of weight. Goals: 1) Reduction of hospitalizations caused by cardiovascular complications by 25% as compared to a control sample. 2) Stable weight (weight ± 5%) in patients with a baseline Body Mass Index >18.5 kg/m^2^
5. Quality of life of the participants will significantly improve. Goals: 1) In the mental subscale of SF-12, 80% of the participants will achieve results that do not differ from norms for the general population. 2) In the physical subscale, 20% will reach results comparable to the general population.
6. Establishment of Standard Operating Procedures (SOPs) including all sectors of the German healthcare system. Finalization of SOPs within 48 weeks of study start.
7. Implementation of an internet-based case file including all sectors of care. Goal: 75% of patients will have used the internet-based case file after 12 months of study start.
8. Implementation and acceptance of telemedicine visits. Goal: 80% of participating physicians will use a telemedicine visit at least once in 80% of their patients within 12 months.

### Evaluation and study analyses

The endpoints will be evaluated by an external independent research institute (IGES Institute GmbH). The study design is a combination of a recurrent institutional cycle design and a multiple time-series design with a non-equivalent control group allowing minimizing bias because of the non-randomized research design of the study [16]. Evaluation will comprise an outcome evaluation for endpoints 1 to 5 and a process evaluation for endpoints 6 to 8 in accordance with the guidelines of the Medical Research Council [17, 18].

The evaluation will use different sources of data: claims data from the statutory health insurances, data assessed during the study on patients’ health, adherence and quality of life as well as data from documentation and process evaluation. Goal 1 (increase of adherence to scheduled follow-up visits) will be evaluated with data from the internet-based case file and with claims data on number of follow-up care visits at the private practice nephrologists and the transplant centers respectively. For Goal 2 (increase of adherence to IS therapy) the evaluation will use IS trough and target levels from the internet-based case file and the BAASIS interview as conducted in the psychosomatic assessments. Goal 3 (reduction of transplant-associated complications) can be evaluated with claims data on hospital utilization and costs as well as on graft loss. The cardiovascular fitness of the patients (Goal 4) will be evaluated with claims data on hospitalizations due to cardiovascular events, with participation rates in training programs as documented in the internet-based case files and with data from the cardiovascular assessments on BMI and on fitness indicators (lactate, heart rate and blood pressure as well as subjective burden). For the evaluation of goal 5 (improvement of quality of life), health-related quality of life measures as assessed in the psychosomatic module will be used. For goals 6 to 8 (implementation and acceptance of SOPs, internet-based case files, telemedicine treatments), data from study documentation on implementation of SOPs, on the internet-based case file and on the telemedicine treatments will be used. In addition, patients as well as private practice nephrologists and nephrologists at the transplantation centers will take part in yearly process evaluations to assess acceptance of all aspects of the program.

To evaluate the goals longitudinal and cross-sectional analytic procedures are used. In both, comparisons between the included patients and the control groups are conducted if applicable. Longitudinal analyses will be conducted with regression analyses in mixed models with repeated measures. This approach is feasible to estimate within-person change over time and group differences in within-person change over time. Cross-sectional group comparisons will be analyzed with regression models without repeated measures. All models will be adjusted for possible confounders, such as age of patients, distance to transplant center or co-morbidities. Descriptive statistics will be conducted to illustrate results, achievement of endpoints (see section on endpoints) and sample characteristics. All effects will be tested with a significance level of *p* = .05.

Group differences in within-person change and levels at one point in time will be evaluated with data available for the comparison groups (mainly data from statutory health insurances). For example, in year one after transplantation costs for hospitalizations of incident patients who received their transplantation in 2017 are expected to be lower as compared to the historical comparison group (prevalent patients with transplantation between 2010 and 2016) and as compared to the external control group (with transplantation in 2017 at different transplant centres). Additionally, data on adherence assessed with the BAASIS interview of prevalent patients who are included in the study 12 months after transplantation can be compared to the adherence scores of incident patients in month 12 after transplantation.

For data of the psychosomatic and cardiovascular assessments, the evaluation of process and health status are assessed in regular visits of the participating incident and prevalent patients: Within-person change after transplantation will be analyzed. For example, we would expect positive changes in quality of life in incident and prevalent patients during the study period. We would also expect an increase in usage and acceptability of telemedicine treatments among the nephrologists.

Selection bias seems unlikely as the study includes all transplantation centres in Lower Saxony and a large majority of nephrologists in private practice is expected to join the study. The participating statutory health insurances cover more than 75% of the population of this federal state. Several important outcome variables are collected in a highly standardized and reliable way as claims data.

### Legal basis and further development of care

The KTx360° study is based on a contract for “special care” with German statutory health insurances according to §140a of the German Social Code, Book Five. The accounting for the nephrologists in private practice will be processed by the Association of Statutory Health Insurance Physicians as in regular care to make the procedures for the participating physicians in private practice as easy as possible. The work of the (pediatric) nephrologists in private practice will be compensated by a three-monthly flat rate per participating patient andextra reimbursement for each telemedicine visit.

With the goals to improve graft survival and to reduce hospitalizations, the KTx360° project will have a considerable positive effect on health care. It can be anticipated that even with moderate clinical effects, efficiency of health care will be improved by both increasing patient quality of life and by reducing health care costs. Additionally, introducing the internet-based patient file will increase efficiency by ensuring regular visits between the transplant center, the patients, and the nephrologists and by reducing travel costs. By developing multisectoral standard operating procedures and implementation of telemedicine, treatment standards of tertiary care centres will be made available in ambulatory care and in rural areas for kidney transplant recipients.

Our model of care can be transferred as a blueprint to transplantation programs in other transplant centers including other organs, to other German federal states and to centers of rare diseases. The multidisciplinary structures including sports medicine and psychosomatic medicine are available in most transplant centers. The innovative eHealth aspects of KTx360° are particularly appropriate for rural areas. The other modules of KTx360° should also be effective in more densely populated regions. KTx360° has been designed to make it transferable to other organs or regions even during the project duration.

## Discussion

This study will provide novel fundamental data whether an integrated care model combining case management, internet-based case files, video consultations/virtual visits, adherence training and physical exercise can reduce patient and graft loss after kidney transplantation and thereby reduce health care costs and improve quality of life. As intended by the German Government, this study has the potential to optimize post-transplant care in Germany and all elements of our study that are evaluated positively, can become part of standard healthcare in Germany. If successful, our program will be extended to other transplanted organs and to all regions of Germany and costs of positively evaluated elements will be covered by public health insurance companies.

## Abbreviations

IS: Immunosuppressants
KTX: Kidney transplantation
